# Stimulation of extracellular invertase production from spent yeast when sugarcane pressmud used as substrate through solid state fermentation

**DOI:** 10.1186/2193-1801-1-81

**Published:** 2012-12-28

**Authors:** Rahul Kumar, Balakrishnan Kesavapillai

**Affiliations:** Technology Business Incubator (TBI), Bannari Amman Institute of Technology, Sathyamangalam, 638 401 India

**Keywords:** Invertase, Pressmud, Spent yeast, SSF, Crude invertase characterization

## Abstract

Efforts were made to utilize the waste/by-product of two agro-process industries namely pressmud from sugar processing industries and spent yeast from distilleries manufacturing ethanol from cane molasses, for the production of microbial invertase. Our experimentation indicated that these two sources could be ideally utilized for the production of invertase through solid substrate fermentation (SSF). SSF with spent yeast had given highest specific activity of 430 U/mg in 72 h of fermentation. Inoculum percentage of yeast cells on pressmud was optimized as 50% (w/w) with a combination inoculum of spent yeast and fresh cultured yeast at a ratio of 7:3. Crude enzyme was characterized for optimum pH and temperature and maximum activity was recorded at pH 5.0 and at a temperature of 40°C. Impacts of metal ions and detergents on invertase action were studied in which Mn^2+^, Fe^3+^, Al^3+^ and detergents had enhanced the activity of the enzyme whereas Cu^2+^ and Zn^2+^ inhibited the enzyme activity. Purification of 9.8 folds was obtained by using three phase partition method.

## Background

Invertase or β-fructofuranosidase (EC 3.2.1.26) resulting in the production of invert sugar which has a lower crystallinity than sucrose at high concentrations, finds numerous applications in the food industry. Confectionary’s preference for invert sugar hovers around its ability to keep the products fresh and soft for prolonged periods. Soluble invertase is used in the sweet industry for the production of artificial honey. Enzyme catalysed hydrolysis has the advantage of colourless products compared to the coloured version obtained through acid hydrolysis (Arica *et al.,*^2000^; Bayramoglu *et al.,*^2003^).

Invertase occurs widely in nature and its presence has been reported in plants, certain animal tissues and microorganisms. There are several reports available inliterature for the purification of invertase from different sources employing various traditional purification processes (Liu *et al.,*^2006^; Guimaraes *et al.,*^2009^; Jegou *et al.,*^2009^; Hussain *et al.,*^2009^; Nguyen *et al.,*^2005^).

Studies have been carried out by using synthetic medium for preparation of invertase while a little attention has been paid on its production from un-conventional inexpensive sources (Vitolo *et al.,*^1995^; Ashokkumar *et al.,*^2001^; Rashad *et al.*, 2006; Guimaraes *et al.,*^2007^; Guimaraes *et al.,*^2009^). Also, the production of enzymes by solid state fermentation (SSF) have potential advantages over submerged fermentation (SmF) with respect to uncomplicatedness in operation, high productivity fermentation, less favorable conditions for growth of contaminants and concentrated product formation (Ashokkumar *et al.,*^2001^).

Growing concern about pollution that occurs from agricultural and industrial wastes has motivated curiosity in converting waste materials into commercially valuable products. The agro-food industry produces large volumes of wastes, both solids and liquids resulting from the production, preparation and consumption of food. Besides their pollution and hazardous aspects, in many cases, food processing wastes might have potential for recycling raw materials or for conversion into useful product of higher value (Sangeetha *et al.,*^2005^; Mamma *et al.,*^2008^; Rashad and Nooman 2008and Guimaraes *et al.,*^2009^).

Under these circumstances it was thought that it would be ideal to scout for desirable sources exhibiting favorable levels of invertase action suitable for commercial exploitation. So the present study deals with the production, partial purification and characterization of one of the useful industrial enzymes (invertase) by utilization of some agro-processing wastes such as sugarcane pressmud and distillery spent yeast. Though there could be more costly, sophisticated and unpredictable solution to arrive at, we presume our methodology of hitting upon a system with untapped residual invertase activity could be the less costly, stable alternative which can be successfully exploited commercially.

## Results and discussion

### Solid state fermentation

#### Optimization of composition of different constituents for SSF

Optimization of SSF for invertase activity using pressmud was attempted in these studies. Tray no 2 holding 10% spent yeast and nutrient mix added to pressmud, had shown higher specific enzyme activity (373.2 U/mg at 72 h; Table 1). There is a preferred release of more invertase with spent yeast samples in the pressmud milieu compared to the cultured yeast (Table 1). The membrane changes that would have taken place when present in the ethanol rich medium for prolonged periods and such stress filled ethanol production milieu would have made the spent yeast cells more susceptible to the stimulation from the SSF culturing on pressmud. There are reports of enzymes getting released from microbial cultures (when grown on solid substrates) as extracellular enzymes which otherwise were intra cellular when grown in liquid cultures (Lekha and Lonsane 1994; Mitchell and Lonsane 1992). Lonsane and Ghildyal (1992) reported this sort of stimulated release with glucose oxidase and invertase earlier.

**Table 1:** **Production of invertase by SSF with different components**

		Specific activity (U/mg)			
Tray No.	Sample	24 hrs.	48 hrs.	72 hrs.	96 hrs.
1	Control	48	47	46	43
2	10% Spent yeast	168	288	373	297
3	10% Culture yeast	210	223	267	240
4	5% Spent yeast + 5% culture yeast	192	283	275	260
5	Same as 3^rd^ tray with 2% sugarcane juice spray at every 12 h	130	186	234	318

Culture yeast with spraying of sugarcane juice (tray 5) at intervals of 12 h had given increase in specific enzyme activity continuously (Table 1) till 96 h. It may be due to adaptability of the culture yeast to steadily start producing invertase in the presence of additional sugar in the spray liquid. Aranda *et al*. (2006) reported that an increase in invertase production noticed when glucose was present in the medium (up to 100 g/L) by *Aspergillus niger* in SSF.It is also suggesting the possibility that Tray 5 (Table 1) gave higher invertase activity at only 96 h owing to the possibility of yeast cells opting to consume reducing sugar available along with sucrose in the sugarcane juice which was sprayed onto the SSF tray intermittently.

#### Optimization of time of harvest for SSF

Fermentation harvest time (72 h) was optimized in SSF with 10% spent yeast as inoculum. A specific enzyme activity of 430 U/mg was reported at the end of 72 h of cultivation (Table 2). Alegre *et al*. (2009) have also reported 72 h as the optimum time for the production of extracellularinvertase at 30°C by *Aspergillus caespitosus.* Similar incubation period (3 days) for both intra and extracellular *A. niger* invertase was reported by Sirisansaneeyakul et al. (2000), Ashokkumar *et al.* (2001) and Mamma et al. (2008), found that the highest invertase activity produced by *A. niger* cultivated on dry orange peels was 72.5 U/g drysubstrate at 72 h, while the highest activity produce by *N. crassa* was 74.0 U/g dry substrate under the same conditions. Rashad and Nooman (2009) had found out the highest productivity of invertase was 272.5 U/g dry substrate in 96 h in red carrot residue by SSF using *Saccharomyses cerevisiae*. Similar behaviour was reported for extracellular invertase production from *A. niger* (Park and Pastores 2003), from *S. cerevisiae* (Rashad *et al.,*^2006^) and from *A. flavus* (Uma *et al.,*^2010^). The enhancement in peak invertase activity reported in Table 2 when compared with that in Table 1 is due to the open tray cultivation adapted as SSF for the experimentation dealt in Table 1 and contained atmosphere tray SSF (tray covered with poly bag) with provision of passive gas exchange for the experiment dealt in Table 2. This means less of other organisms intimidating with SSF in the contained culturing. The specific activity at 96 h was low (Table 2) which could be due to accumulation of heat during exothermic activity in poly bag contained fermentation.

**Table 2:** **Optimization of duration for SSF**

Time (hrs.)	12	24	36	48	60	72	84	96
Specific invertase activity detected in Pressmud + 10% Spent yeast system (U/mg)	67.1	146.3	227.3	295.4	390.9	**430.8**	255.8	86.2

#### Optimization of % inoculum for SSF

Inoculum percentage in SSF was optimized as 50% (w/w; i.e. 50 g of spent yeast pellet added to 100 g of pressmud with nutrient mix and water added remain the same as in other trays with varied levels of spent yeast used as inoculum) with specific enzyme activity of 395.2 U/mg. When there are still enhanced levels of inoculum, contamination with fungus was noticed (Table 3). Increasing the percentage inoculum of washed spent yeast cells on pressmud gave enhanced levels of invertase till 50% (w/w) inoculum. Beyond this level, the trend was reversed and invariably there was fungal growth observed in the plates. This may be due to the dead yeast being used as substrate by the fungus present in the unsterilized pressmud and due to less promising growth rate and activity spectrum of introduced yeast cells whenin excess, due to contact inhibition of cells. Humidity enhancement in the microenvironment also would have favoured fungal contaminations at higher levels of inoculum added.

**Table 3:** **Optimization of inoculum percentage for SSF**

**Inoculum%(Spent yeast)**	10	20	30	40	**50**	60	70	80	90	100
**Specific activity (U/mg)**	298.6	312.7	349.1	368.6	**395.2**	381.7	322.8	242.1	267.3	272.9

#### Optimization of inoculums composition i.e. ratio of Spent yeast and culture yeast

Different composition of inoculum also influenced the yield of enzyme and hence the enzyme activity. The ratio (7:3) of spent yeast and culture yeast had given high specific enzyme activity of 297.3 U/mg for the total 20% inoculum (Table 4).

**Table 4:** **Optimization of inoculums composition i.e. ratio of Spent yeast and culture yeast**

**Spent yeast : Culture yeast (together they constituted 20% (w/w) of pressmud**	1:9	3:7	5:5	**7:3**	9:1
**Specific activity (U/mg)**	147.3	192.7	247.5	**297.3**	289.7

### Characterization of crude invertase

#### Optimum pH determination and optimum temperature determination

**Figure 1:**
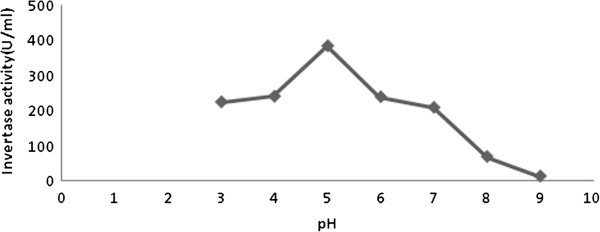
**The effect of optimum pH (5.0) on the activity of invertase.**

**Figure 2:**
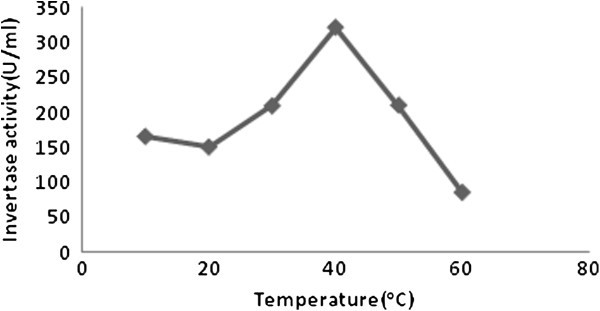
**The effect of optimum temperature (40°C) on activity of invertase.**

Crude invertase extracted from pressmud combinations was found possessing an optimum pH of 5.0 (Figure 1). The optimum temperature obtained for the crude enzyme activity was 40°C (Figure 2). Sanjay and Sugunan (2006) reported maximum activity at pH 5 and at 50°C. Andjelkovic *et al.* (2010) reported as 3.5- 5.0 the optimum pH. The optimum pH from 3.5 to 7.0 hasbeen reported for invertases isolated from different yeasts (Belcarz *et al.*, 2002; Persike *et al.,*^2002^). Andjelkovic *et al.*^2010^) reported as 60°C as optimum temperature of invertase action. In general, invertases show high activity in the temperature range of 35–75°C depending to their sources andalso incubation time (Persike *et al.,*^2002^; Kern *et al.,*^1992^). Nguyen *et al.* (2005) reported the optimum temperature of *A. niger* invertase as 50°C. Higher values of optimum invertase temperatureswere reported by many authors (Rubio *et al.,*^2002^; Guimaraes *et al.,*^2007^; Hussain *et al.,*^2009^), while lower value (30°C) was reported by (Rashad *et al.*^2006^).

#### Impacts of metal ions and detergents

**Figure 3:**
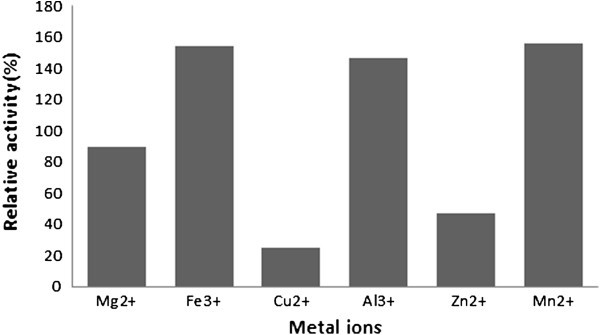
**The effect of metal ions (5 mM) on activity of invertase.**

Enhancement of crude invertase activity was observed with Mn^+2^ and Fe^+3^. Cu^+2^showed inhibitory effects for the crude invertase enzyme activity. Small increase in the activity was observed with detergents also (Figure 3). (Dahot & Noomrio 1996) reported increase in invertase I and II activities in the presence of MnCl_2_, CoCl_2_ and CaCl_2_ but the same completelyinhibited with EDTA due to chelation with metal ions. Rashad and Nooman (2009) indicated that the enzyme was completely inhibited by Hg^2+^ at low concentration (1 mM), while it was slightly inhibited by Ba^2+^, Zn^2+^, and Fe^2+^ at the same concentration. On the other hand, a slight increase inthe enzyme activity was noticed by using 1 mM of Co^2+^. The inhibition of invertase by Hg^2+^ was reported by many authors (Guimaraes *et al.,*^2009^; Rashad *et al.,*^2006^; ^2001^) and they suggested that thiolgroups at the catalytic site are important for the invertase activity. Stimulation of invertase activity by Co^2+^wasalso reported by Rubio *et al.* (2002) and Rashad *et al.* (2006), while (Nguyen *et al.*^2005^) found that the enzyme was slightly inhibited by addition of 1 mM Co^2+^.

#### Purification of crude invertase by Three Phase Partition method (TPP)

The overall purification of invertase from culture yeast was done by TPP which gave 9.8 fold of purification for aqueous phase with specific enzyme activity of 3388 U/mg (Table 5). Our findings on the suitability of TPP method for the purification of invertase was supported by a number of findings. Akardere *et al.* (2010) had found 15 fold of purification for aqueous phase from Baker’s yeast by TPP method. Ozer *et al.* (2010) had found 8.6 fold of purification for interfacial phase from tomato by TPP method. A TPP process was reported as giving purification folds of 9 for the interphase of tomato pectinase (Sharma and Gupta 2001).

**Table 5:** **Summary of overall purification of invertase by three-phase partitioning**

Steps	Total activity (Unit)	Total protein (mg/100 ml)	Specific activity(U/mg)	Purification fold
Crude extract	165	48	344	1
TPP-aqueous phase	62	1.83	3388	9.85

## Conclusions

The present study though confirmed the presence of invertase enzyme activity in fresh Pressmud and spent yeast, residual specific invertase activity from spent yeast when cultured on fresh pressmud was found to be higher in titers. Solid state fermentation triggered on press mud as substrate gave maximum specific activity at 72 h of spent yeast cultivation. Combination of spent yeast & fresh cultured yeast (7:3) as inoculum gave maximum invertase activity at 72 h. Till 50% w/w of spent yeast on pressmud favored maximum invertase production. The crude enzyme activity was optimized in terms of temperature and pH. Impact studies for the metal ions showed fair increase in the residual enzyme activity in presence of metal ions and marginal increase by detergents. About 10 folds of purification of crude enzyme activity obtained by means of three phase partition method.

## Methods

### Microorganisms

Spent yeast samples (yeast cell slurry deposited at the bottom of ethanol fermentation tanks inoculated with *Sachharomyces cereviceae*) were provided by Bannari Amman Distilleries, Modur, and Erode. The slurry collected was centrifuged at lab level (6000 rpm for 10 min) and the resultant cell pellet was washed twice with water to discard chemical residues settled along with cells. This washed spent yeast cells were introduced to pressmud at 10% (w/w) (if there was variation in inoculum percentage, the same was specifically mentioned) as inoculum.

A local isolate of *S. cereviceae* maintained in YDP slants was used as fresh cultured yeast (2 loops of the yeast transferred to 50 mL of YDP broth held in 250 mL conical flask and incubated at 120 rpm for 24 h) wherever added as part of the inoculum. This culture was maintained as glycerol stock in glycerol: water (1:1) mix at −20°C. Sub cultures were made by streaking on to YDP agar.

### Solid state fermentation (SSF) of sugarcane pressmud

#### Optimization of composition of different constituents for SSF

Fresh pressmud (100 g each) was taken in five different sterilized stainless steel plates (30×20 cm) and different compositions of constituents were added to carryout SSF in open (as in experiment dealt in Table 01)/closed and contained environment with a controlled temperature and humidity of 30°C; 94% respectively. In tray number one (un-inoculated plate, pressmud was mixed with nutrient mix containing sucrose (1%), ammonium sulphate (0.4%) and peptone (0.2%) dissolved in 25 mL of tap water. The composition of media constituents in various trays was depicted below (Table 6). The contents of the plates were mixed well and kept for solid state fermentation for a period of 96 h. Humidity (90-95%) was maintained by manually spraying of sterile distilled water in every 12 h. Sample (20 g) were collected at every 12 h andfermented solid was mixed with the 80 ml of extraction buffer (100 mM Potassium phosphate, 10 mMβ-mercaptoethanol and 1 mM phenyl methyl sulfonyl fluoride) and kept for stirring for 30 min at 10°C. Leachate was filtered with nylon cloth and centrifuged at 6000 rpm for 15 min at 10°C. Supernatant was used as crude invertase and went for invertase assay.

**Table 6:** **Production of invertase by SSF with different components**

Tray No.	Pressmud, g	Water, mL	Nutrient mix	Inoculum (w/w)
1	100	25	Added	No inoculum
2	100	25	Added	10% spent yeast
3	100	25	Added	10% culture yeast
4	100	25	Added	5% spent yeast and 5% culture yeast
5	100	25	Added	Same as 3^rd^ tray with 2% sugarcane juice spray at every 12 h

#### Optimization of duration of fermentation time

In 100 g of fresh pressmud, 10% of spent yeast (w/w) mixed with nutrient mix in 25 ml of tap water was added. They were mixed well and spread like a bed on a stainless steel tray and kept for solid state fermentation for 96 h. Humidity (90-95%) was maintained by manually spraying water in every 12 h. Samples (20 g each of fermented solids) were collected at every 12 h and they were processed as mentioned above.

#### Optimization of Spent yeast inoculum for SSF

In 100 g of fresh pressmud, different amounts of spent yeast, from 10 g -100 g were mixed along with nutrients in 25 ml of tap water. They were mixed well and spread like beds on stainless steel trays. SSF carried out as mentioned above.

#### Optimization of inoculum composition i.e. ratio of Spent yeast and culture yeast

For every 100 g of fresh pressmud 20% of inoculum was mixed with nutrients in 25 ml of tap water. Inocula contained different ratio of spent yeast and culture yeast (1:9, 3:7, 5:5, 7:3 and 9:1). SSF carried out as explained above.

#### Determination of residual Invertase activity and enzyme concentration

Crude invertase activity of the samples was estimated as per the method described earlier by Bernfeld (1955). One unit of enzyme activity corresponds to the release of 1 μM of glucose in one minute, by 1 mL of enzyme under the assay conditions.

#### Estimation of protein in Solid state fermentation leachate

The protein content of lechate samples was quantified spectrophotometrically at 595 nm according to Bradford (1976) method for all samples using bovine serum albumin as standard.

### Characterization of crude invertase

#### Optimum pH determination

In order to determine the optimum pH of the crude enzyme preparation from SSF, 0.2 M Glycine-HCl, 0.2 M Acetate, 0.2 M Sodium phosphate and 0.2 M Tris–HCl buffers were used in the pH range of 3.0, 4.0-5.0, 6.0-8.0 and 9.0 respectively. All the assays were done at a constant temperature of 35°C.

#### Optimum temperature determination

For determination of the optimum temperature, enzyme activity was assayed at different temperatures in the range from 10°C to 60°C. The desired temperature was provided by using a water bath. The enzyme assays were carried out using 50 mM acetate buffer (pH 4.7).

#### Impacts of metal ions and detergents

To determine the effects of metal ions on the crude invertase obtained as SSF leachate, 5 mM concentrations of the following metal ions- Al^+3^, Ca^+2^, Cu^+2^, Fe^+3^, Mg^+2^, Mn^+2^& Zn^+2^ and detergents - SDS and TritonX-100 were added into the reaction mixture separately. The enzyme activities were measured; a test sample in the absence of metal ions was used as control.

#### Purification of crude invertase by Three Phase Partition method (TPP)

TPP method as reported earlier by Akardere *et al.* (2010) was used for the partial purification of the crude enzyme sample.
